# Hyperoside, a Flavonoid Compound, Inhibits Proliferation and Stimulates Osteogenic Differentiation of Human Osteosarcoma Cells

**DOI:** 10.1371/journal.pone.0098973

**Published:** 2014-07-01

**Authors:** Ning Zhang, Mei-Dan Ying, Yong-Ping Wu, Zhi-Hong Zhou, Zhao-Ming Ye, Hang Li, Ding-Sheng Lin

**Affiliations:** 1 Department of Orthopaedics, 2nd Affiliated Hospital, School of Medicine, Zhejiang University, Hangzhou, P.R. China; 2 Institute of Pharmacology and Toxicology, College of Pharmaceutical Sciences, Zhejiang University, Hangzhou, P.R. China; 3 Department of Orthopaedics, 3rd Affiliated Hospital, Zhejiang Chinese Medical University, Hangzhou, P.R. China; 4 Department of Orthopaedics, Wenzhou Medical College-Affiliated Second Hospital, Wenzhou, P.R. China; Colorado State University, United States of America

## Abstract

Osteosarcoma, one of the most common malignant bone tumours, is generally considered a differentiation disease caused by genetic and epigenetic disruptions in the terminal differentiation of osteoblasts. Novel therapies based on the non-cytotoxic induction of cell differentiation-responsive pathways could represent a significant advance in treating osteosarcoma; however, effective pharmaceuticals to induce differentiation are lacking. In the present study, we investigated the effect of hyperoside, a flavonoid compound, on the osteoblastic differentiation of U2OS and MG63 osteosarcoma cells *in vitro*. Our results demonstrated that hyperoside inhibits the proliferation of osteosarcoma cells by inducing G_0_/G_1_ arrest in the cell cycle, without causing obvious cell death. Cell migration assay further suggested that hyperoside could inhibit the invasion potential of osteosarcoma cells. Additionally, osteopontin and runt-related transcription factor 2 protein levels and osteocalcin activation were upregulated dramatically in hyperoside-treated osteosarcoma cells, suggesting that hyperoside may stimulates osteoblastic differentiation in osteosarcoma cells. This differentiation was accompanied by the activation of transforming growth factor (TGF)-β and bone morphogenetic protein-2, suggesting that the hyperoside-induced differentiation involves the TGF-β signalling pathway. To our knowledge, this study is the first to evaluate the differentiation effect of hyperoside in osteosarcoma cells and assess the possible potential for hyperoside treatment as a future therapeutic approach for osteosarcoma differentiation therapy.

## Introduction

Osteosarcoma is the most common non-haematological malignancy of bone in children and adults [1]. Despite modern treatment protocols that combine chemotherapy, surgery and sometimes radiotherapy, the 5-year survival rate for patients diagnosed with osteosarcoma has remained at 60–70% since the 1970s [2], [3]. Osteosarcoma is associated with poor prognosis due to its high incidence of metastasis and chemoresistance. Although surgical techniques and implants have shown steady improvement, current chemotherapeutic agents seem wholly similar to those used forty years ago, with significant morbidity including cardiac toxicity, infertility and renal dysfunction [4]–[6]. As >80% of osteosarcomas are poorly differentiated histopathologically, novel therapies based on the non-cytotoxic induction of cell differentiation-responsive pathways could represent a significant advance.

Differentiation therapy is a therapeutic modality aimed at re-activating endogenous differentiation programs in cancer cells with subsequent cellular maturation of the tumour and concurrent loss of the tumour phenotype. In the era before retinoic acid- based differentiation therapy for acute promyelocytic leukaemia (APL), various cytotoxic chemotherapies caused a progressive improvement in remission rates from 50 to 80%, of which ∼35% had predicted long-term survival. However, with the current use of retinoic acid and chemotherapy, more than 90% of patients with newly diagnosed APL achieve complete remission, and about 75% are cured [7], [8]. The application of differentiation therapy for solid tumours has been hindered by the absence of developmental models of cancer progression that correlate cancer subtypes to stages of normal development. Osteosarcoma cells share many similar features to undifferentiated osteoprogenitors including a high proliferative capacity, resistance to apoptosis and similar expression profiles of many osteogenic markers such as connective tissue growth factor, runt-related transcription factor 2 (RUNX2), alkaline phosphatase (ALP), osterix and osteocalcin [9], [10]. Increasing evidence suggests that osteosarcoma cells can be induced to mature osteoblasts by certain compounds, including retinoic acid [11]. However, no clinical applications for differentiation therapy in patients with osteosarcoma have been reported to date. Therefore, the identification of substances capable of inducing the osteogenic differentiation of osteosarcoma cells is currently receiving considerable attention.

**Figure 1:**
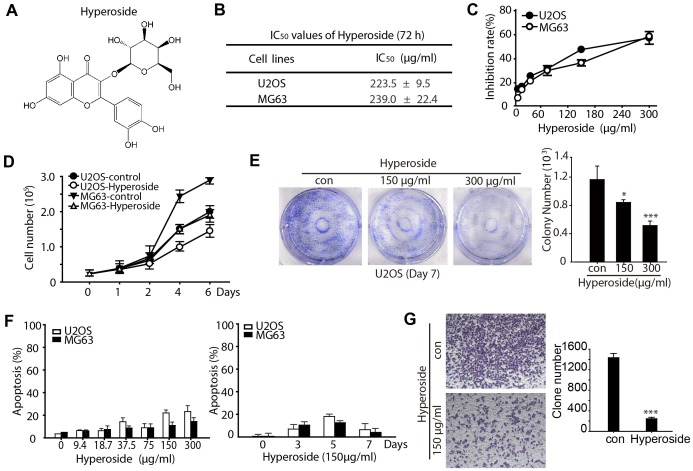
Hyperoside inhibits the proliferation of osteosarcoma cells. (a) Structural formula of hyperoside. (b) U2OS and MG63 cells in 96-well plates were treated with serial concentrations of hyperoside for 3 days and the IC_50_ was determined using the sulforhodamine B (SRB) assay. (c) U2OS and MG63 cells were treated with serial concentrations of hyperoside for 3 days and proliferation was determined by SRB assay. (d) U2OS and MG63 cells were treated with 150 µg/ml hyperoside for 0–6 days and proliferation was determined by SRB assay. (e) U2OS cells in six-well plates were treated with the indicated concentrations of hyperoside for 7 days, and viable clones were stained with crystal violet. (f) U2OS and MG63 cells were treated with serial concentrations of hyperoside for indicated days and apoptosis was determined by flow cytometry. (g) U2OS cells were seeded into the top chamber of transwell plates and treated with hyperoside for 24 h. Cells that had migrated (on the bottom side of the filter) were stained with crystal violet and counted by light microscopy.

Numerous studies have shown that some Chinese medicines can induce osteosarcoma differentiation [12], [13]. Flavonoids are plant polyphenols found in vegetables, fruit and beverages of plant origin, and are well known for their physiological antipyretic, analgesic and anti-inflammatory activities [14]. Hyperoside (also called quercetin 3-O-β-d-galactoside; Fig. 1A), a major pharmacologically active component from hypericumperforatum [15], exerts multiple bioactivities, including myocardial protection [16], anti-redox [17] and anti-inflammatory activities [18]. However, no studies have investigated the application of hyperoside in treating osteosarcoma.

In this study, we hypothesise that hyperoside inhibits the proliferation of osteosarcoma cells *in vitro* and induces G_0_/G_1_ arrest. Furthermore, we hypothesise that hyperoside induces osteoblastic differentiation of osteosarcoma cells, and that the transforming growth factor (TGF)-β signalling pathway may contribute to hyperoside-induced osteoblastic differentiation. By inducing differentiation and inhibiting proliferation, hyperoside may be aa potent agent of differentiation therapy for treating osteosarcoma.

## Materials and Methods

### Cells and reagents

Human osteosarcoma cell line U2OS and MG63 were purchased from the Shanghai Institute of Biochemistry and Cell Biology (Shanghai, China). U2OS and MG63 cell lines were cultured in RPMI-1640 or DMEM media plus 10% heat-inactivated FBS and incubated at 37°C in a 5% CO_2_ atmosphere. Penicillin (100 U/ml) and streptomycin (100 U/ml) were added in the medium.

Hyperoside was purchased from Zelang Biological Technology Co., Ltd. (Nanjing, China), and was dissolved in dimethyl sulfoxide (DMSO).

### Cellular proliferation analysis

The anti-proliferation activity of hyperoside treatment was measured by SRB assay or determine by manual count using a standard hemocytometer following trypan blue staining. U2OS and MG63 cells were plated at 3000 cells per well in a 96-well plate, allowed to adhere overnight, and treated with hyperoside for 3 days. After treatment, cells were fixed with 10% trichloroacetic acid for 1 h at 4°C, washed with deionized water and stained by incubating with 0.4% SRB dye for 15 min at room temperature. Then the cells were washed with 1% acetic acid and the bound SRB dye was solubilized with 10 mmol/L unbuffered Tris. The optical density was measured at 540 nm using a plate reader. For growth curve, cells were treated with hyperoside for indicated time, and total number were determined by manual counting in a standard hemocytometer following trypan blue exclusion.

### Colony Formation Assay

U2OS cells were plated at 1000 cells per well in a 6-well plate, allowed to adhere overnight, and treated with the indicated concentrations of Hyperoside continuously. After 7 days of incubation, the cells were stained with 0.2% crystal violet after methanol fixation, and the numbers of colonies containing more than 50 cells were counted.

### Cell migration assay

Cell migration assay was performed in 24-well Transwell plates (8.0 µm, pore size). U2OS cells were seeded into the top chamber of transwell plates and 150 µg/ml hyperoside were added to the lower chambers in 600 µl medium. After 24 hours at 37°C, the upper sides of the filters were carefully washed with PBS, and cells remaining on the upper faces were removed with a cotton wool swab. Transwell filters were then fixed and stained using crystal violet. Cells that had migrated (on the bottom side of the filter) were counted using light microscopy. The average number of migrating cells per field was assessed by counting at least four random fields per filter. Each experiment was done in duplicate.

### Cellular apoptosis and Cell-Cycle Analysis

The proportion of apoptotic cells and cells in each cell-cycle phase were determined by flow cytometry measurement of DNA content. U2OS and MG63 cells were treated with hyperoside for 0–7 days. Then cells were harvested, fixed in 75% ethanol overnight at −20°C, and incubated in 0.5 mL of propidium iodide staining solution (50 mg/mL RNase A and 50 mg/mL propidium iodide) at room temperature for 30 min. The cellular DNA content was analyzed on a FACS Calibur flow cytometer using the Cell Quest Pro software (BD Biosciences, San Jose, CA). The percentage of each population was measured using ModFIT software (BD Biosciences). At least 20 000 cells were analyzed for each data point.

### Western blotting

Western blotting was conducted as reported previously [19]. Briefly, proteins were quantified using the DC Protein Assay kit. After protein quantification and normalization, equivalent amounts of proteins were electrophoresed on 8 to 15% SDS-PAGE gels and transferred to PVDF membranes (Millipore, Bedford, MA). After incubation with primary antibodies, the proteins were visualized by incubation with HRP-conjugated secondary antibodies followed by enhanced chemiluminescence detection (Biological Industries, BeitHaemek, Israel). The OPN polyclonal antibody (FL-314), RUNX2 polyclonal antibody (M-70), β-Actin polyclonal antibody (C-11) were obtained from Santa Cruz Biotechnology Inc. (Santa Cruz, CA). The P21 Waf1/Cip1 polyclonal antibody (12D1), P27Kip1 polyclonal antibody (D69C12) were purchased from Cell Signaling Technology. The density analysis was performed for each WB band by using Quantity One 1-D analysis software (Bio-Rad Laboratories), and the average relative intensity value was shown along with the corresponding bands.

### Real-Time Quantitative PCR Assay

Total RNA was prepared using the Trizol reagent (cat # BS409, Bio Basic Inc.) as recommended by the manufacturer. cDNA was synthesized using 2 µg of total RNA with random hexamer primers and RevertAidTM M-MuLV Reverse Transcriptase (Fermentas International, Inc., Burlington, Ontario, Canada). Equilibrated amounts of cDNA were taken for transcript PCR amplification. Quantitative PCR analysis was performed with the Eppendorf epGradient Mastercycler according to the standardized thermal profile of the system set previously by the manufacturer with QuantiTectTM SYBR Green PCR kits (Qiagen Inc.) for detection. The sequences of the primers used for the PCR were as follows: OPN: forward 5′-TCACAGCCATGAAGATATGCTGG-3′, reverse5′-TACAGGGAGTTTCCATGAAGCCAC-3′; RUNX2: forward 5′-CCAGCCACCTTTACTTACAC-3′, reverse5′-AGCGTCAACACCATCATTCT-3′; Osteocalcin: forward: 5′-GTGCAGCCTTTGTGTCCAAG-3′, reverse 5′-TCAGCCAACTCGTCACAGTC-3′; TGF-β: forward5′- CAACAATTCCTGGCGATACC-3′, reverse 5′-GAACCCGTTGATGTCCACTT-3′; BMP-2 forward 5′-AAAACGTCAAGCCAAACACAAA-3′, reverse 5′-GTCACTGAAGTCCACGTACAAAGG-3′; GAPDH: forward 5′-GTC ATC CAT GAC AAC TTT GG-3′, reverse 5′-GAG CTT GAC AAA GTG GTC GT-3′. Relative expression levels of the target genes were normalized with the control gene GAPDH.

### Immunofluorescence

Cells grown and treated in 96-well plates were fixed with 4% formaldehyde in PBS for 15 min, washed two times with PBS, and blocked with 5% FBS and 1% Triton X-100 in PBS for 1 h at room temperature. Osteocalcin were detected with antibody (SC-74495) from Santa Cruz Biotechnology Inc. and PE-conjugated secondary antibody diluted 1∶100 in PBS for 2 h at room temperature. Cells were then washed three times in PBS and imaged with a Leica DMI 400B fluorescence microscope.

### Statistical Analysis

All data were reported as mean ± standard deviation (SD). Normality tests were performed before comparing the data. One-way analysis of variance (ANOVA) was used to determine the difference of mRNA expression, the apoptotic percentage,the clony number, the proliferation and inhibition assay, with the post–hoc Tukey test to compare between groups when significant variation was found. A cutoff of P<0.05 applied for statistical significance. All statistical analyses were performed with SPSS version 16.

## Results

### Hyperoside inhibits cell proliferation without cytotoxicity in human osteosarcoma cells

To determine the optimal hyperoside concentration required to inhibit proliferation in human osteosarcoma cells, serial concentrations of hyperoside were assessed in U2OS and MG63 cell lines using the sulforhodamine B (SRB) assay. As shown in Fig. 1B and 1C, hyperoside (0–300 µM) inhibited cell proliferation in U2OS and MG63 cells in a dose-dependent manner at 72 h, with IC_50_ values of 223.5 and 239.0 µM, respectively. This anti-proliferation effect of hyperoside remained similar when using different concentrations of serum (2%, 5% and 10%) (Fig. S1A). Compared to the corresponding control groups, 150 µg/ml hyperoside inhibited proliferation in a time-dependent manner in both U2OS and MG63 cells (Fig. 1D). Moreover, this inhibition occurred without inducing cell death, as evaluated by trypan blue staining (data not shown). Therefore, we used 150 µg/ml hyperoside in subsequent experiments involving both cell lines. The colony-formation assay further supported the conclusion that hyperoside inhibited U2OS cell proliferation (Fig. 1E). Moreover, Annexin V-propidium iodide (PI) staining followed by flow cytometry analysis confirmed that there is only a little increase the percentage of apoptotic or necrotic U2OS and MG63 cells after serial concentrations of hyperoside treatment for 3 days or 150 µg/ml hyperoside treatment for 0–7 days (Fig. 1F). ROS detection together with the western blotting results of PARP and pro caspase 3 also indicated that 150 µg/ml hyperoside treatment could not induce obvious apoptosis (Fig. S1B, S1C). Cell migration assays suggested that hyperoside inhibited the invasion potential of osteosarcoma cells *in vitro* (Fig. 1G). These results revealed that hyperoside significantly inhibits the proliferation of osteosarcoma cells.

### Hyperoside induces G0/G1 arrest of osteosarcoma cells

**Figure 2:**
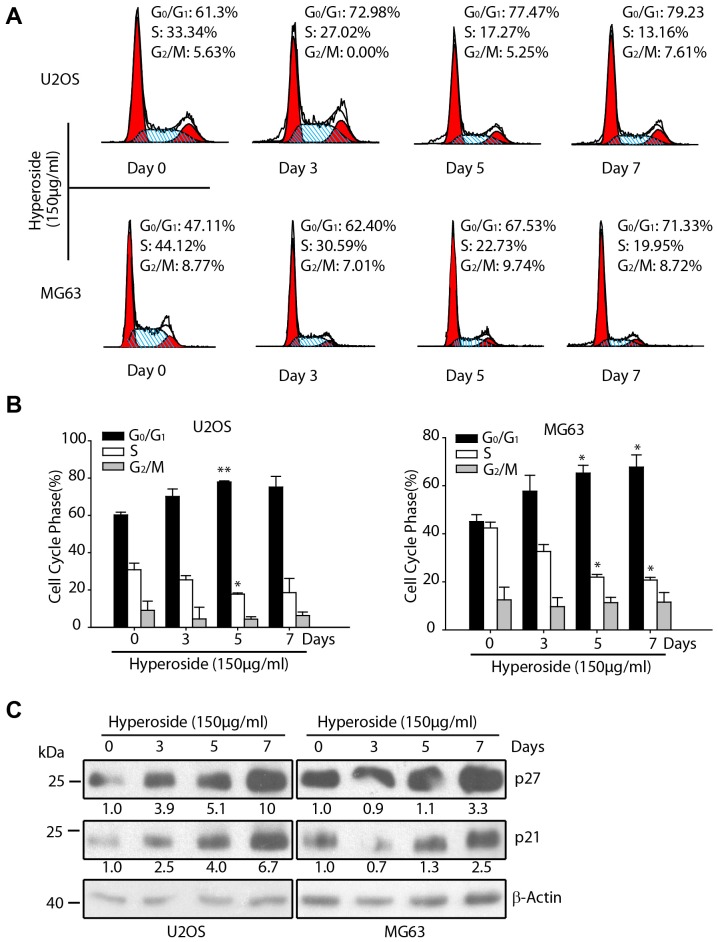
Hyperoside induces G0/G1 arrest of osteosarcoma cells. (a) U2OS and MG63 cells were treated with 150 µg/ml hyperoside for 0–7 days and the cell cycle phase was detected using a FACSCalibur flow cytometer. The percentages of G_0_/G_1_ cells are indicated. (b) Statistical representation of data from Fig. 2a. (c) U2OS and MG63 cells were treated with 150 µg/ml hyperoside for 0–7 days. Then, cells were lysed and immunoblotted using anti-p27, anti-p21 and anti-β-actin antibodies. The numbers below the p21 and p27 indicated the density of each WB band.

G1 exit is a critical stage in the cell cycle in which cells commonly commit to differentiation [20], [21]. Thus, we investigated the effect of hyperoside on the G_0_/G_1_ phase of the cell cycle in osteosarcoma cells. U2OS and MG63 cells were treated with 150 µg/ml hyperoside for 0–7 days, and the cell cycle distribution was determined by PI staining of the collected cells followed by flow cytometry analysis. As illustrated in Fig. 2A, hyperoside treatment induced an increased proportion of cells in G_0_/G_1_ in a time-dependent manner, with an accompanying decrease in the number of cells in the S phase. Whereas 61.3% of untreated U2OS cells were in the G_0_/G_1_ phase, 72.98% of the hyperoside-treated U2OS cells were in the G_0_/G_1_ phase on day 3 and 79.23% on day 7 (Fig. 2B, upper panel). A similar result was obtained in the MG63 osteosarcoma cell line, in which 71.33% of the cells were in G_0_/G_1_ after 7 days of hyperoside treatment, compared to 47.11% in the control group (Fig. 2B, lower panel). Fig. 2B shows the statistical representation of Fig. 2A. Moreover, Western blotting showed dramatic increases in the protein levels of p21 and p27 [22]—two critical cell cycle inhibitors—in hyperoside-treated cells in a time-dependent manner (Fig. 2C), indicating that p21 and p27 are involved in hyperoside-induced G_0_/G_1_arrest. These results suggested that the inhibition of proliferation in osteosarcoma cells results from hyperoside-induced cell cycle G_0_/G_1_ arrest.

### Hyperoside stimulates osteogenic differentiation of osteosarcoma cells

**Figure 3:**
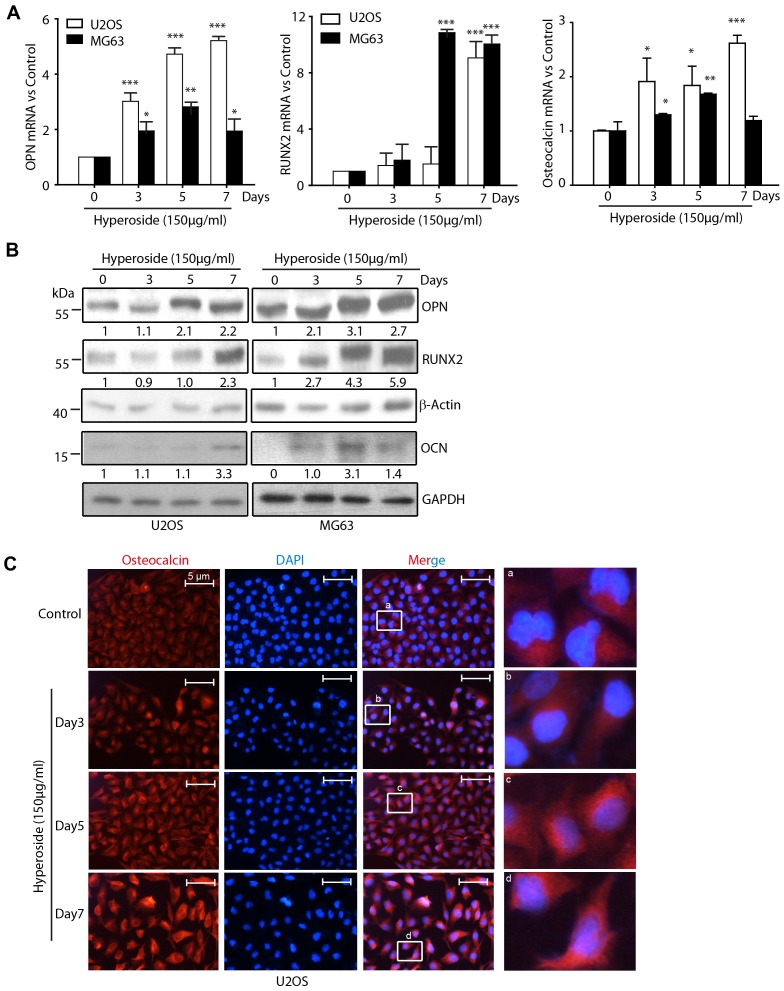
The differentiation is occurred after osteosarcoma cells treating with Hyperoside. (a) U2OS and MG63 cells were treated with 150 µg/ml hyperoside for 0–7 days. Total RNA were extracted and subjected for the amplification of the indicated transcripts by Real Time PCR. Expression levels were normalized to GAPDH. (b) U2OS and MG63 cells were treated with 150 µg/ml hyperoside for 0–7 days. The whole cell lysates were obtained and fractionated on a 10% SDS-PAGE gel. Next, an immuneblotting assay with anti-OPN, anti-RUNX2, anti-osteocalcin (OCN) or anti-β-Actin antibodies was performed to determine the levels of OPN, RUNX2 and osteocalcin. The numbers below the bands indicated the density of each WB band. (c) U2OS cells were treated with hyperoside for the indicated times. Immunofluorescence analysis of osteocalcin (red) expression was performed. DNA was stained with a fluorescent dye, DAPI.

Previous studies have shown that osteoblast differentiation is characterised by the synthesis of osteopontin (OPN), RUNX2 and osteocalcin [23], [24]. Therefore, we investigated whether these markers were upregulated in hyperoside–treated osteosarcoma cells. U2OS and MG63 cells were treated with 150 µg/ml hyperoside for 0–7 days, and OPN, RUNX2 and osteocalcin expression levels were determined by real-time PCR and Western blotting. Fig. 3A shows that the mRNA levels of OPN, RUNX2 and osteocalcin in both cell types were upregulated significantly in hyperoside-treated cells in a time-dependent manner. Moreover, the protein expression levels of OPN, RUNX2 and osteocalcin were increased dramatically (Fig. 3B) in a manner consistent with the changes in mRNA levels. We further determined the expression of osteocalcin using an immunofluorescence assay. As Fig. 3C illustrates, osteocalcin expression (red signal) was increased dramatically in U2OS cells treated with 150 µg/ml hyperoside for ∼3–7 days. Therefore, the increased OPN, RUNX2 and osteocalcin expression demonstrated that differentiation occurred of osteosarcoma cells, which might be stimulated by hyperoside. Although the magnification times of all the rows are completely same, since the reviewer has different opinion about our magnification, we have measured all the width of the nuclei in 4 groups and calculated the average width of nuclei (Fig. 3C). As shown in Fig. S2, the average width of nuclei in control, day 3, day 5, and day 7 groups are 0.84±0.13, 0.82±0.17, 0.73±0.11, and 0.92±0.14 µm. This result indicated that although there are a little bit difference in the width of nuclei in different groups, there is no statistical significant.

### Hyperoside upregulates TGF-beta and BMP2 expression

**Figure 4:**
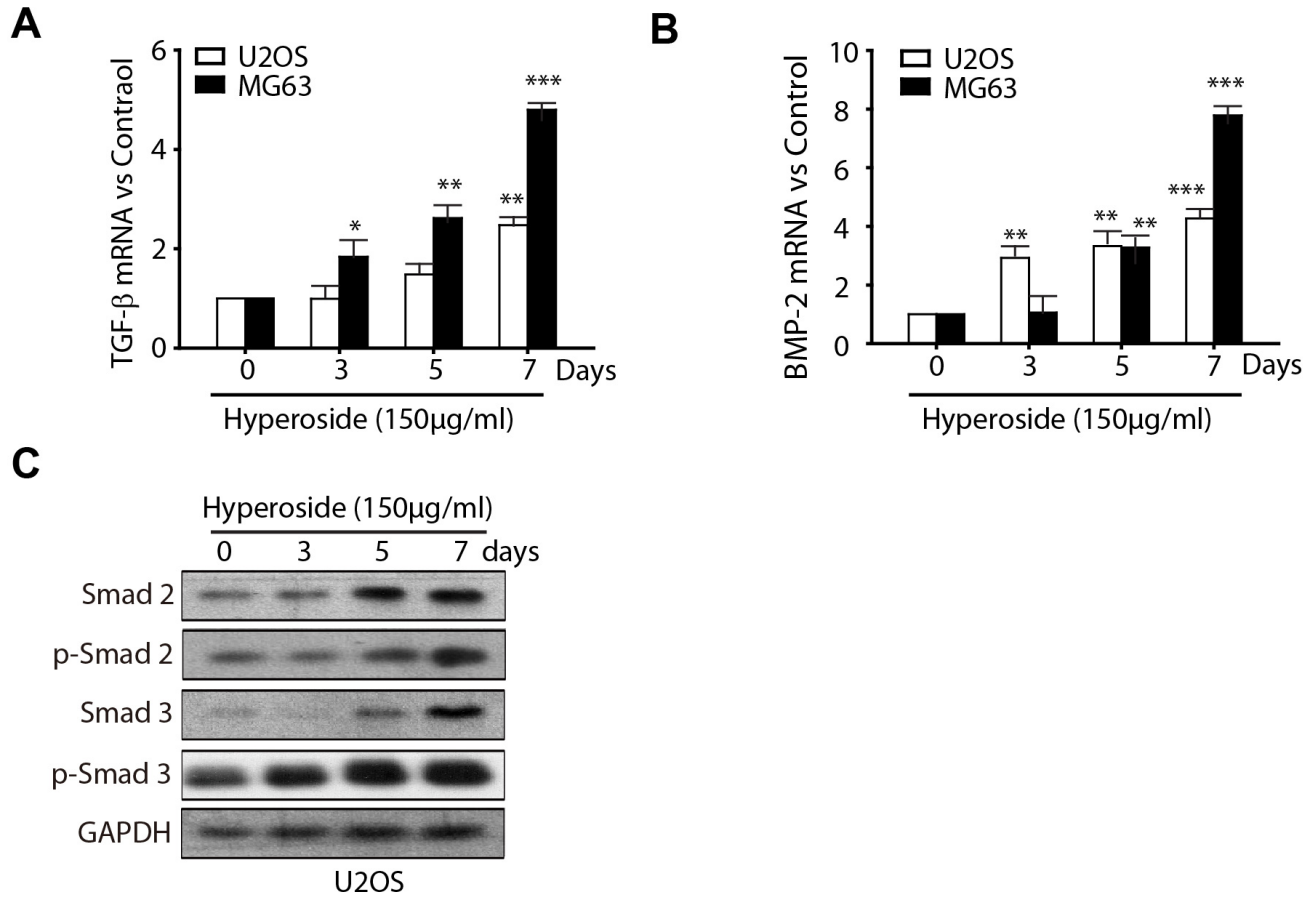
TGF-β signalling contributes to hyperoside-induced osteoblastic differentiation. (a, b) U2OS and MG63 cells were treated with 150 µg/ml hyperoside for 0–7 days. Total RNA was extracted and subjected to real-time PCR amplification of the indicated transcripts. Expression levels were normalised to those of GAPDH. (c) U2OS cells were treated with 150 µg/ml hyperoside for 0–7 days, and an immunoblotting assay using an anti-Smad2, anti-p-Smad2, anti-Smad3, anti-p-Smad3 or anti-GAPDH antibody was performed.

TGF-β, a protein that controls proliferation, cellular differentiation and other functions in most cells, has been identified as a constituent of the bone matrix [25]. Previous studies have shown that TGF-β, combined with 1,25-dihydroxyvitamin D3, promotes differentiation of the MG63 human osteosarcoma cells [26]. Bone morphogenetic protein 2 (BMP2) belongs to the TGF-β superfamily of proteins. Like other bone morphogenetic proteins, BMP2 plays an important role in the development of bone and cartilage [27]. To further gain insight into the molecular signalling regulating the differentiation effect of hyperoside, we further examined the changes of TGF-β and BMP2 signalling. As illustrated in Fig. 4A, TGF-β mRNA levels were increased significantly in hyperoside-treated U2OS and MG63 cells. A significant increase in BMP2 mRNA levels was also observed in our study. The downstream events of TGF-β signalling were further investigated after hyperoside exposure in U2OS cells. The expression of Smad2 and Smad3 (substrates for TGF-β) were increased in a time-dependent manner after hyperoside treatment, and the phosphorylation levels of Smad2 and Sma3 were also increased (Fig. 4C). Taken together, our data suggest that TGF-β signalling is involved in hyperoside-induced osteoblastic differentiation of osteosarcoma cells.

## Discussion

Although preoperative and postoperative chemotherapies have improved the 5-year survival rate, the prognosis for osteosarcoma remains poor [1], highlighting the urgent need for new and improved therapies. The induction of differentiation may represent a promising alternative to conventional chemotherapy for certain malignancies [11]. For example, the success of all-trans retinoic acid (ATRA)-based differentiation therapy in APL has led researchers to apply differentiation-based approaches to other AML subtypes. Nevertheless, most current chemotherapies and radiation therapies target the rapidly proliferating tumour cells, with little consideration for promoting tumour cell differentiation. It is conceivable that a combined therapeutic approach targeting proliferation and differentiation phases of tumour cells would be more efficacious and less prone to inducing chemoresistance.

This study provided evidence that hyperoside inhibits proliferation and induces G_0_/G_1_ arrest of osteosarcoma cells. Hyperoside induces osteoblastic differentiation of osteosarcoma cells. Although the specific mechanism underlying hyperoside-mediated osteoblastic differentiation of osteosarcoma cells remains undefined, these results suggest that hyperoside treatment represents a promising future therapeutic strategy. Genetic and/or molecular changes in osteoprogenitors may disrupt the osteogenic differentiation pathway, leading to osteosarcoma development. Thus, recent investigations have focused on the therapeutic potential to overcome differentiation defects associated with osteosarcoma and thereby prevent tumourigenesis. Such therapies include nuclear receptor agonists, growth factors and transcription factors [28]–[31]. Another factor that should be mentioned is that, although our data suggest that hyperoside shifts cells into an osteogenic lineage, proliferation never totally ceases. Either not all the cells are induced to terminal differentiation by hyperoside or even when expressing osteoblast markers, the cells are continuing to proliferate. Longer time period experiments are required to determine whether cells stop proliferating, and what pepercent, or whether hyperoside slows the proliferation rate.

Recent findings suggest that only a small fraction of transformed cells—known as cancer stem cells (CSCs)—are capable of reconstituting the diverse cell types within a particular tumour [32], [33]. Studies focusing on CSC characterisation have reported that some subpopulations of osteosarcoma cells express prospective CSC markers, including CD117^+^, Stro-1^+^ [34] or CD133^+^ [35]–[37]. Hyperoside apparently inhibited the proliferation of the U2 and MG63 cells in this study, but the data are insufficient to make any conclusions about the effects on CSCs. Therefore, further studies are needed to address this issue.

Although the multiple biological functions of hyperoside have attracted increasing attention, the bioavailability and tissue distribution of hyperoside remains unclear. In general, the bioavailability of flavonoids is relatively low due to limited absorption and rapid elimination. It is reported that the plasma concentrations of total metabolites ranged from 0 to 4 µmol/L with an intake of 50 mg aglycone equivalents depending on the flavonoids [38]. Therefore, researchers made many efforts to enhance the oral bioavailability of flavonoids, such as prodrugs of scutellarin [39], phospholipid complex of silybin [40], and proliposome of silymarin [41]. Our results demonstrate that hyperoside could induce osteoblastic differentiation of osteosarcoma cells without cytotoxicity, thus hyperoside could serve as a naturally derived tumour differentiation agent. However, efforts to improve the efficiency *in vivo* are required urgently.

In summary, our results show that the differentiation is occurred *in vitro* when osteosarcoma cells treated with hyperoside, a flavonoid compound derived from a traditional Chinese medicine. As hyperoside induces differentiation activity of osteosarcoma cells *in vitro*, future studies should determine the hyperoside-induced differentiation activity of osteosarcoma cells *in vivo*. Our findings support the possibility of the application of flavonoid compounds in differentiation therapy for osteosarcoma, which might lead to new treatment strategies that use differentiation-based approaches for the treatment of osteosarcoma.

## Supporting Information

Figure S1 (a) Hyperoside inhibits the proliferation of osteosarcoma cells cultured in the presence of various serum concentrations. MG63 cells were treated with serial concentrations of hyperoside for 3 days and cultured in the presence of 2, 5 or 10% serum. Proliferation was determined by SRB assay. (b) U2OS and MG63 cells were treated with serial concentrations of Hyperoside or cisplatin for 0–12 h. ROS levels were measured by FACS using carboxy-DCFDA. (c) U2OS and MG63 cells were treated with 150 µg/ml hyperoside for 0–7 days, and an immuneblotting assay with anti-pro caspase 3, anti-PARP, or anti-GAPDH antibodies were performed. The numbers below the bands indicated the density of each WB band.
(TIF)

Figure S2 The width of nuclei in osteosarcoma cells. U2OS cells were treated with hyperoside for the indicated times, and the average width of nuclei were measured.
(TIF)
