# Supplementary figures and images for: Hyperoside, a Flavonoid Compound, Inhibits Proliferation and Stimulates Osteogenic Differentiation of Human Osteosarcoma Cells
Source: PLoS One. 2014 Jul 1;9(7):e98973. doi: 10.1371/journal.pone.0098973 (PMC4077650; doi:10.1371/journal.pone.0098973)

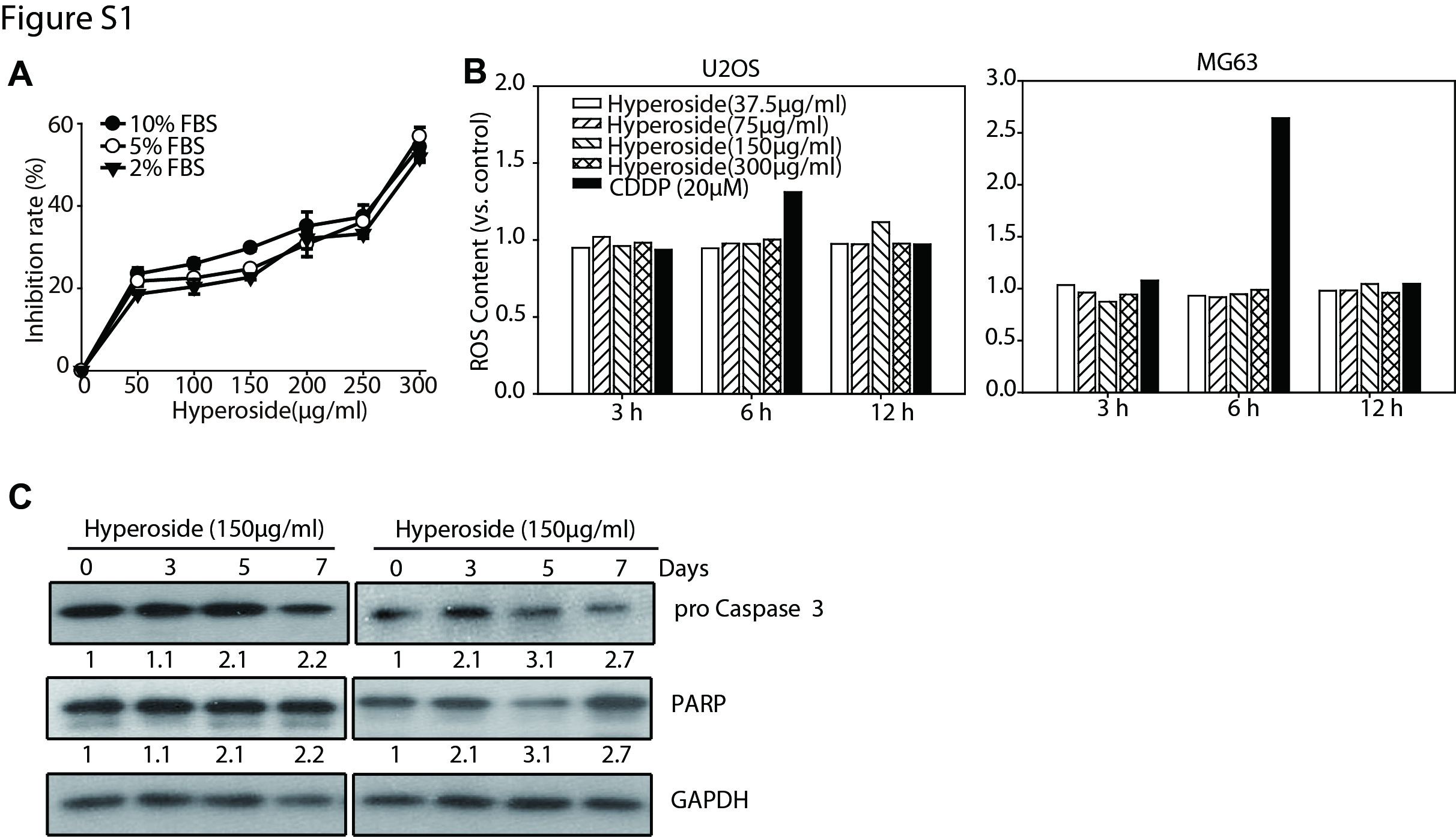

Supplement: Figure S1 — (a) Hyperoside inhibits the proliferation of osteosarcoma cells cultured in the presence of various serum concentrations. MG63 cells were treated with serial concentrations of hyperoside for 3 days and cultured in the presence of 2, 5 or 10% serum. Proliferation was determined by SRB assay. (b) U2OS and MG63 cells were treated with serial concentrations of Hyperoside or cisplatin for 0–12 h. ROS levels were measured by FACS using carboxy-DCFDA. (c) U2OS and MG63 cells were treated with 150 µg/ml hyperoside for 0–7 days, and an immuneblotting assay with anti-pro caspase 3, anti-PARP, or anti-GAPDH antibodies were performed. The numbers below the bands indicated the density of each WB band. (TIF) [file pone.0098973.s001.tif]

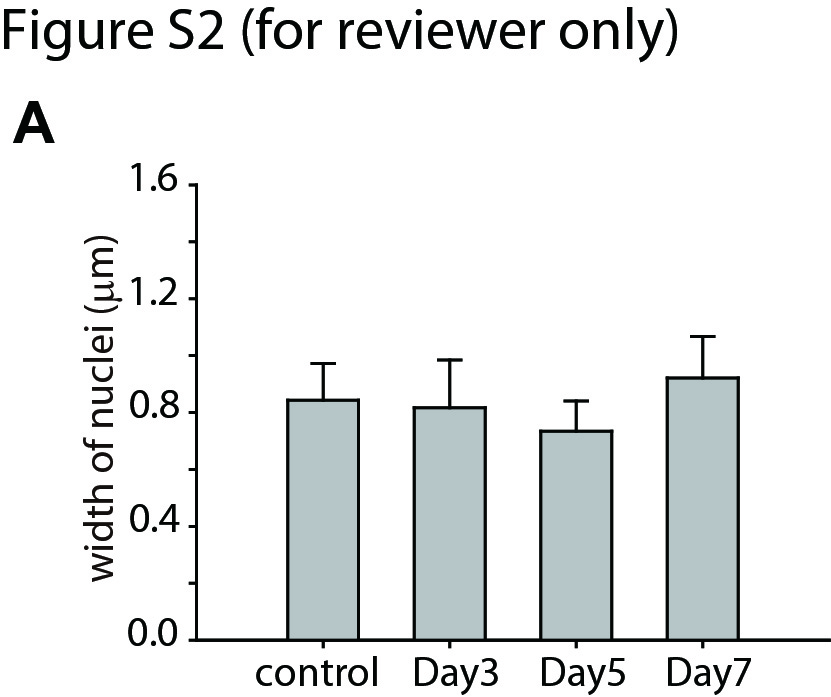

Supplement: Figure S2 — The width of nuclei in osteosarcoma cells. U2OS cells were treated with hyperoside for the indicated times, and the average width of nuclei were measured. (TIF) [file pone.0098973.s002.tif]
